# Evaluation of a Meropenem and Piperacillin Monitoring Program in Intensive Care Unit Patients Calls for the Regular Assessment of Empirical Targets and Easy-to-Use Dosing Decision Tools

**DOI:** 10.3390/antibiotics11060758

**Published:** 2022-06-02

**Authors:** Ferdinand Anton Weinelt, Miriam Songa Stegemann, Anja Theloe, Frieder Pfäfflin, Stephan Achterberg, Franz Weber, Lucas Dübel, Agata Mikolajewska, Alexander Uhrig, Peggy Kiessling, Wilhelm Huisinga, Robin Michelet, Stefanie Hennig, Charlotte Kloft

**Affiliations:** 1 Department of Clinical Pharmacy and Biochemistry, Institute of Pharmacy, Freie Universitaet Berlin, Kelchstr. 31, 12169 Berlin, Germany; ferdinand.weinelt@fu-berlin.de (F.A.W.); lucas.95@gmx.net (L.D.); franz.weber@fu-berlin.de (F.W.); robin.michelet@fu-berlin.de (R.M.); stefanie.hennig@certara.com (S.H.); 2 Graduate Research Training Program PharMetrX, Freie Universitaet Berlin/Universität Potsdam, 12169 Berlin, Germany; 3 Department of Infectious Diseases and Respiratory Medicine, Charité-Universitaetsmedizin Berlin, Corporate Member of Freie Universitaet Berlin, Humboldt-Universitaet zu Berlin, Berlin Institute of Health, 13353 Berlin, Germany; miriam.stegemann@charite.de (M.S.S.); frieder.pfaefflin@charite.de (F.P.); stephan.achterberg@charite.de (S.A.); agata.mikolajewska@charite.de (A.M.); alexander.uhrig@charite.de (A.U.); 4 Antimicrobial Stewardship, Charité-Universitaetsmedizin Berlin, Corporate Member of Freie Universitaet Berlin, Humboldt-Universitaet zu Berlin, Berlin Institute of Health, 13353 Berlin, Germany; 5 Pharmacy Department, Charité-Universitaetsmedizin Berlin, Corporate Member of Freie Universitaet Berlin, Humboldt-Universitaet zu Berlin, Berlin Institute of Health, 13353 Berlin, Germany; anja.theloe@charite.de; 6 Labor Berlin—Charité Vivantes GmbH, 13353 Berlin, Germany; peggy.kiessling@laborberlin.com; 7 Institute of Mathematics, Universität Potsdam, Karl-Liebknecht-Str. 24-25, 14476 Potsdam, Germany; huisinga@uni-potsdam.de; 8 School of Clinical Sciences, Faculty of Health, Queensland University of Technology, Brisbane, QLD 4000, Australia; 9 Certara, Inc., Princeton, NJ 08540, USA

**Keywords:** meropenem, piperacillin/tazobactam, antimicrobial stewardship, critically ill, antibiotics, pharmacokinetic/pharmacodynamic

## Abstract

The drug concentrations targeted in meropenem and piperacillin/tazobactam therapy also depend on the susceptibility of the pathogen. Yet, the pathogen is often unknown, and antibiotic therapy is guided by empirical targets. To reliably achieve the targeted concentrations, dosing needs to be adjusted for renal function. We aimed to evaluate a meropenem and piperacillin/tazobactam monitoring program in intensive care unit (ICU) patients by assessing (i) the adequacy of locally selected empirical targets, (ii) if dosing is adequately adjusted for renal function and individual target, and (iii) if dosing is adjusted in target attainment (TA) failure. In a prospective, observational clinical trial of drug concentrations, relevant patient characteristics and microbiological data (pathogen, minimum inhibitory concentration (MIC)) for patients receiving meropenem or piperacillin/tazobactam treatment were collected. If the MIC value was available, a target range of 1–5 × MIC was selected for minimum drug concentrations of both drugs. If the MIC value was not available, 8–40 mg/L and 16–80 mg/L were selected as empirical target ranges for meropenem and piperacillin, respectively. A total of 356 meropenem and 216 piperacillin samples were collected from 108 and 96 ICU patients, respectively. The vast majority of observed MIC values was lower than the empirical target (meropenem: 90.0%, piperacillin: 93.9%), suggesting empirical target value reductions. TA was found to be low (meropenem: 35.7%, piperacillin 50.5%) with the lowest TA for severely impaired renal function (meropenem: 13.9%, piperacillin: 29.2%), and observed drug concentrations did not significantly differ between patients with different targets, indicating dosing was not adequately adjusted for renal function or target. Dosing adjustments were rare for both drugs (meropenem: 6.13%, piperacillin: 4.78%) and for meropenem irrespective of TA, revealing that concentration monitoring alone was insufficient to guide dosing adjustment. Empirical targets should regularly be assessed and adjusted based on local susceptibility data. To improve TA, scientific knowledge should be translated into easy-to-use dosing strategies guiding antibiotic dosing.

## 1. Introduction

Over the last decades, the rate of new antibiotic drugs being approved for clinical use has considerably slowed while bacterial resistance against frequently used antibiotic drugs has continued to increase [1,2,3]. To tackle the spread of antimicrobial resistance, the World Health Organization introduced a draft for a ‘Global Framework for Development and Stewardship to Combat Antimicrobial Resistance’ in 2017 [4]. One key objective of the framework is the optimisation of antimicrobial drug use to improve individual clinical outcomes and to minimise the worrying spread of antimicrobial resistance as an unintended consequence of antimicrobial use. To achieve this objective, the framework recommends the implementation of local antimicrobial stewardship (AMS) programs to monitor and improve antibiotic use.

The purpose of these local AMS programs is the systematic and recurrent evaluation of the local antimicrobial use and the implementation of coordinated interventions designed to promote the optimal use of antimicrobial agents. A critical element in optimal antimicrobial use is the selection of the optimal antibiotic drug and, equally important, the optimal dosing based on relevant pharmacokinetic (PK) and pharmacodynamic (PD) principles [5,6,7,8].

To attain the desired antibiotic exposure in an individual patient, the administered dosing regimen needs to be selected based on the PK properties of the administered antibiotic drug, patient-specific characteristics, and the susceptibility of the causative pathogen. Especially in critically ill patients, pathophysiological changes can lead to altered PK and exposure of antibiotic drugs [9]. As a consequence, antibiotic drug exposures outside the target range are frequently observed, increasing the risk of therapy failure in this vulnerable patient population [10,11,12,13,14,15]. At the same time, frequent antibiotic therapy and associated higher proportions of resistant pathogens are commonly observed in intensive care unit (ICU) patients [16,17]. Suboptimal antibiotic exposure and overprescribing of antibiotics in this patient population could be seen as an additional accelerator of the spread of antimicrobial resistance [5]. Routine monitoring of antibiotic drug concentrations followed by subsequent dosing adjustments based on the observed concentrations and predefined target concentrations represents one possible intervention local AMS programs can implement to improve antimicrobial use [18]. 

Two broad spectrum beta-lactam antibiotics, meropenem and piperacillin, the latter in fixed combination with the beta-lactamase inhibitor tazobactam, are among the most commonly used antibiotics to treat infections in ICU patients [19,20]. The bactericidal activity of both originates from the inactivation of penicillin-binding proteins and the resulting inhibition of the bacterial cell wall synthesis [21,22]. Current evidence supports that their antimicrobial activity is linked to the time period of the free drug concentration exceeding the minimum inhibitory concentration (MIC) of a pathogen (*f*T_>MIC_) [23,24]. Frequently, the target exposure in ICU patients is defined as the free drug concentrations remaining above the MIC for 100% of the dosing interval (100% *f*T_>MIC_), i.e., minimum drug concentration at the end of the dosing interval just before the next infusion above the MIC [25]_._ At the same time, targets as high as 100% *f*T_>4×MIC–8×MIC_ have been recommended for both drugs to achieve effective treatment and to reduce antibiotic resistance [26,27]. However, in clinical practice the MIC value of the infecting pathogen and consequently the drug exposure target for the antibiotic therapy is often unknown. In a recent survey, more than half (54%) of the responding clinicians in German ICUs reported not to obtain any MIC results from their laboratories [28]. Even if MIC values are reported by default, commonly neither the infecting pathogen nor its MIC value is available at the start of antibiotic therapy. Consequently, patients infected by an unidentified pathogen have to be treated empirically. During this empirical therapy, pathogen unspecific PK/PD breakpoints or susceptibility thresholds provided by international consortia such as the European Committee on Antimicrobial Susceptibility testing (EUCAST) are targeted instead of the unknown MIC value of the infecting pathogen [29]. In addition to linking exposure to antibiotic efficacy, drug exposure linked to adverse events needs to be considered for an effective and safe antibiotic therapy. For beta-lactam antibiotics, high minimum drug concentrations (meropenem > 64 mg/L, piperacillin > 157 mg/L) have been associated with neurotoxicity and worse outcomes in critically ill patients [26]. Meropenem and piperacillin/tazobactam are primarily renally eliminated via passive glomerular filtration and active tubular secretion via organic anion transporters 1 and 3, with the majority of both drugs being excreted unmetabolised (fraction of unchanged drug excreted in urine: 68% for piperacillin and 50–75% for meropenem) [30,31]. Pathophysiological changes are commonly observed in ICU patients. With regard to the kidneys, renal impairment can be caused by chronic kidney disease or acute kidney injury and augmented renal clearance due to hyperdynamic states [32]. These and other changes have the potential to decrease or increase the renal elimination of meropenem and piperacillin/tazobactam compared to renally healthy individuals. As a consequence, it is recommended to adjust the dosing of meropenem and piperacillin/tazobactam for the renal function of an individual patient [26]. 

In 2019, a drug concentration monitoring program for meropenem and piperacillin/tazobactam was initiated as part of a coordinated intervention of the AMS program at Charité-Universitaetsmedizin Berlin, a tertiary care centre with more than 3000 in-patient beds. The implemented drug concentration monitoring program entailed the determination of antibiotic drug concentrations and MIC values. The analysis of this program aimed to evaluate meropenem and piperacillin/tazobactam dosing in ICU patients by assessing (i) the adequacy of locally selected empirical targets compared to local susceptibility data, (ii) if dosing is adequately adjusted for the renal function and the individual target of each patient, and (iii) if—after drug concentrations become available—dosing is adjusted in target attainment (TA) failure.

## 2. Results

### 2.1. Patient Characteristics

From January 2019 to August 2020, 375 meropenem samples and 230 piperacillin samples were collected from 108 and 96 ICU patients, respectively. For 19 (5%) blood samples taken for meropenem measurements and 14 (6%) blood samples taken for piperacillin measurements, the plasma drug concentrations could not be determined by the laboratory due to delays in sample transportation. For both drugs, the patients included in the study were predominantly male (meropenem: 64.8%, piperacillin/tazobactam: 63.5%), aged over 60 years (median: meropenem: 62.0 years, piperacillin/tazobactam: 65.0 years) and had a median weight of 76.0 kg. Creatinine clearance, serum albumin concentrations, and location of infection were similar for patients receiving meropenem and piperacillin/tazobactam (Table 1). Patients receiving meropenem were found to be more severely ill, with increased sepsis-related organ failure assessment (SOFA) and acute physiology and chronic health evaluation APACHE scores (median score meropenem vs. piperacillin/tazobactam: SOFA: 8 vs. 6, APACHE: 23 vs. 20) and a higher proportion with extracorporeal organ support (percentage of samples during organ support meropenem vs. piperacillin/tazobactam: renal replacement therapy (RRT): 38.1% vs. 23.0%, extracorporeal membrane oxygenation (ECMO): 8.8% vs. 5.22%).

### 2.2. Antimicrobial Treatment

The monitored meropenem dosing regimens prior to the concentration measurements were diverse: In most cases (63.5%), 2 g meropenem were administered as loading dose, while in the remaining cases, it was 1 g meropenem. Administered meropenem maintenance doses included 0.5, 1, 2, 3, 6, and 8 g, with 1 g (50.2%) and 2 g (40.7%) being the two most frequently administered doses. The majority of doses (92.7%) were administered as prolonged infusions over 3 h (31.2%) or 4 h (61.5%) and a further 7.26% as continuous infusion. The most common dosing interval for meropenem was 8 h (77.0%), followed by 6 h (11.0%), 24 h (8.52%), and 12 h (3.15%).

Piperacillin/tazobactam dosing regimens monitored during the study did not substantially vary between patients regarding the administered dose, infusion duration, and dosing interval. For piperacillin, almost all administered doses (98.6% of loading and 99.5% of maintenance doses) consisted of 4 g in combination with 0.5 g tazobactam (others: 1 g piperacillin/0.125 g tazobactam and 2 g piperacillin/0.25 g tazobactam). The majority (98%) of doses were administered as prolonged infusion over 3 h (34.3%) or 4 h (63.7%); the others were prolonged infusions over 2 h (1.5%) or short infusions over 0.5 h (0.5%). The most common dosing interval was 8 h (72.4%), followed by 6 h (18.6%), 12 h (8.54%), and 24 h (0.5%). 

### 2.3. Pathogen Susceptibility 

For 49.1% of patients receiving meropenem and 34.4% of patients receiving piperacillin/tazobactam, the MIC value of the pathogen causing the infection could be determined and was available for the attending physician approximately two days after therapy start (median time after therapy start: meropenem 2.1 days, piperacillin 2.3 days). The majority of observed pathogens was susceptible to the administered antibiotics (meropenem: 93.3%, piperacillin: 97.0%) and most observed MIC values (meropenem: 90.0%, piperacillin: 93.9%) were found to be lower than 8 mg/L for meropenem and 16 mg/L for piperacillin, which represented the lower threshold of the target range set by the AMS team for empirical antibiotic therapy. For meropenem, 75% of the pathogens with a determined MIC value had an MIC value 32 times smaller than the lower threshold of the defined empirical target range. For piperacillin, more than one-fifth (21.2%) of the determined MIC values were within a two-fold deviation and 93.9% within a four-fold deviation from the selected empirical target. An overview of the observed MIC values independent of their pathogen is presented in Table 2.

### 2.4. Target Attainment Assessment

Of the 356 meropenem samples with a reported drug concentration, 127 (35.7%) were within the targeted concentration range, while 49 (13.8%) were below and 180 (50.6%) were above. One in forty (2.5%) meropenem concentration measurements exceeded concentrations linked to an increased risk for neurotoxicity. For piperacillin, 109 of 216 (50.5%) measured drug concentrations were in the targeted concentration range, were 32 (14.8%) below, and 75 (34.7%) were above. One in ten (10.2%) piperacillin concentrations exceeded the toxicity threshold. 

**Figure 1:**
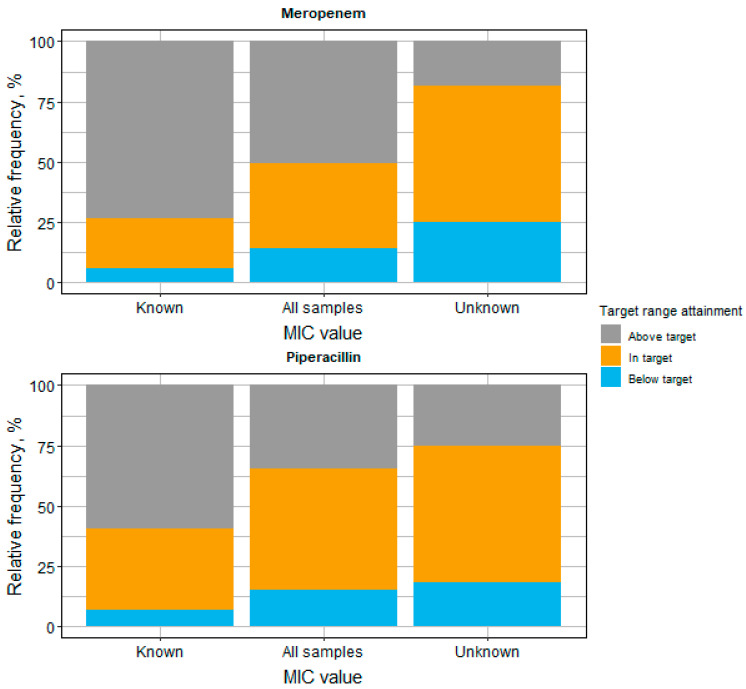
Target range attainment for meropenem and piperacillin stratified by the availability of susceptibility data (MIC value) for the pathogen causing the infection. Target range for the minimum drug concentration defined as 1–5 × MIC. If no MIC values could be determined, an empirical target of 8–40 mg/L and 16–80 mg/L was used for meropenem and piperacillin, respectively. Abbreviations: MIC: minimum inhibitory concentration.

Concentration measurements from patients without a determined MIC value for the causative pathogen (i.e., empirical therapy) had a higher proportion of target attainment than measurements with a determined MIC value (meropenem 56.8% vs. 20.6%, piperacillin 57.0% vs. 33.9%; Figure 1). 

### 2.5. Target Attainment Assessment in Different Renal Function Groups

**Figure 2:**
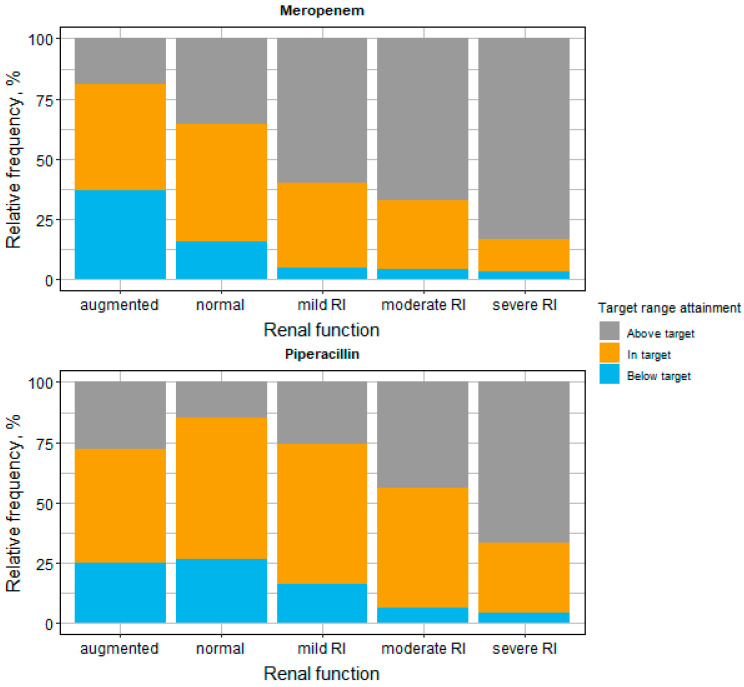
Target range attainment for meropenem and piperacillin stratified by renal function. Target range for the minimum drug concentration defined as 1–5 × MIC. If no MIC values could be determined, an empirical target of 8–40 mg/L and 16–80 mg/L was used for meropenem and piperacillin/tazobactam, respectively. Abbreviations: MIC: minimum inhibitory concentration; RI: Renal impairment.

**Figure A1:**
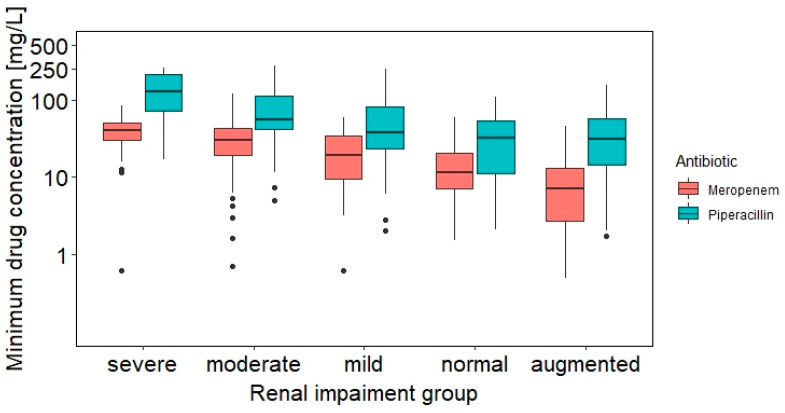
Minimum meropenem concentrations by renal function group. Boxes: interquartile range (IQR), horizontal line: median, whiskers: range below first or above third quartile to 1.5-fold the IQR, points: concentrations outside whiskers.

The observed target attainment significantly differed between patients with different renal functions (*p*-value < 0.01 for both drugs). Patients with severe renal impairment had a higher proportion of samples above the target (target attainment meropenem: 2.78% below, 13.9% in target, 83.3% above; target attainment piperacillin: 4.17% below, 29.2% in target, 66.7% above) compared to patients with augmented renal clearance (target attainment meropenem: 36.9% below, 44.0% in target, 19.0% above; target attainment piperacillin: 25.0% below, 47.2% in target, 27.8% above). The highest frequency of target attainment for both drugs was found in patients with ‘normal’ renal function (RF) (49.2% for meropenem, 58.8% for piperacillin; Figure 2). For both antibiotics, the observed minimum drug concentrations increased with higher impairment of renal function (Appendix A, Figure A1).

### 2.6. Drug Concentration Assessment between Different Target Range Groups 

**Figure A2:**
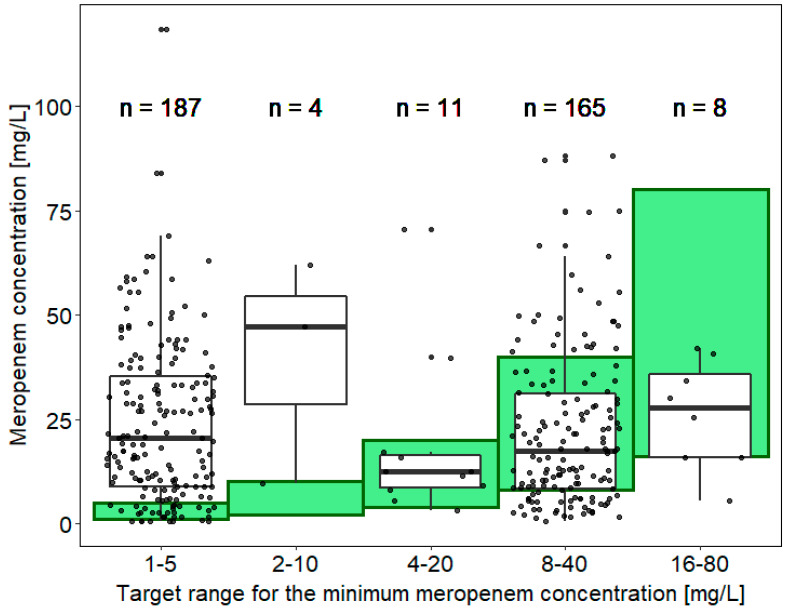
Boxplot of measured meropenem concentrations stratified by targeted drug concentration range. Target range (Green shaded areas) for the minimum drug concentration defined as 1–5 × MIC. If no MIC values could be determined an empirical target of 8–40 mg/L was used. Points: measured meropenem concentrations.

**Figure A3:**
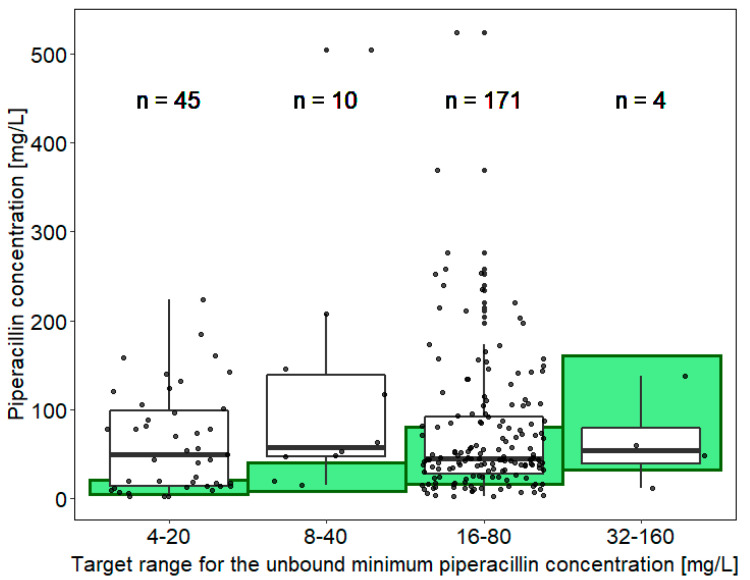
Boxplot of measured piperacillin concentrations stratified by targeted drug concentration range. Target range (Green shaded areas) for the minimum drug concentration defined as 1–5 × MIC. If no MIC values could be determined an empirical target of 16–80 mg/L was used. Unbound piperacillin concentrations were calculated based on the literature reported fraction unbound of 91% in critically ill patients [60]. Points: measured piperacillin concentrations.

Neither for meropenem nor for piperacillin did the measured drug concentrations stratified by their targeted concentration range differ significantly. A boxplot of the observed drug concentrations per targeted concentration range and drug can be found in the Appendix A (Figure A2 and Figure A3). 

### 2.7. Dosing Adaptations

After drug concentration measurement, dosing was adjusted in 6.18% and 4.63% of cases for meropenem and piperacillin, respectively. While the majority of meropenem dosing adaptations were conducted via a change in the administered dose (68.2%), piperacillin dosing was predominantly adjusted by modifying the dosing interval (90%) (Table 3). No dosing adaptations were observed for piperacillin after samples in the target range. The overall proportion of dose adaptations after samples outside versus in the target range did not significantly differ for meropenem (*p*-value = 0.8) but differed for piperacillin (*p*-value = 0.005). 

## 3. Discussion

The antibiotic monitoring program conducted in two ICUs provided two key insights into current dosing practice for the two frequently used antibiotics meropenem and piperacillin: Currently, neither the local pathogen susceptibility data is comprehensively exploited for PK target selection nor is the relationship between renal function and antibiotic clearance for dosing decisions. This leads to poor target attainment (only 4 out of 10 meropenem samples and 5 out of 10 piperacillin samples were found to be in the target range). Hence, the regular assessment of PK targets and easy-to-use dosing decision tools are warranted. 

At the two ICUs at Charité-Universitaetsmedizin Berlin, the MIC of a causative pathogen was determined for only 49.1% of patients receiving meropenem and 34.4% of patients receiving piperacillin/tazobactam. The value was available to the attending intensive care physician approximately two days after the initiation of antimicrobial therapy. This finding corresponds well with those reported by Esteve-Pitarch et al. [34] and once again highlights the need for safe and effective dosing strategies prior to pathogen and MIC detection. To determine if an antibiotic drug is the right choice for a safe and effective therapy, it is important to closely monitor the local susceptibility to the antibiotic. Fortunately, in our analysis the high number of pathogens susceptible to the investigated drugs confirmed that both drugs are suitable for empirical antimicrobial therapy. However, the question arises if the selected targets for empirical therapy were appropriate. For both drugs, more than 90% of the determined MIC values were found to be lower than the lower threshold of the selected empirical target range. Nevertheless, the conclusion based on the assessment of the selected empirical targets differed between meropenem and piperacillin/tazobactam due to two considerations: (i) the percentage of samples without determined MIC values and (ii) the fold difference between the observed MIC values and the selected empirical targets. First, for patients treated with meropenem, the corresponding MIC value of the pathogen was available more often compared to piperacillin (49.1% vs. 34.4%). As a consequence, the reliability that the collected susceptibility data represented the underlying MIC value distribution at the two intensive care wards was higher for meropenem. Second, the lower threshold of the empirical target range was 32 times higher than 75% of MIC values observed for meropenem, while more than a fifth (21.3%) of the determined MIC values for piperacillin were within a two-fold deviation and 93.9% were within a four-fold deviation from the selected empirical target. Given the high uncertainty in MIC value determination [35], this deviation of the observed susceptibility to piperacillin from the selected empirical target was judged to be too low to justify a target reduction for piperacillin. Detecting more causative pathogens, including a concomitant MIC determination, would help to further elucidate the local piperacillin susceptibility and will hopefully increase the reliability of the selected empirical target in the future. For meropenem adjusting, i.e., reducing the empirical target from 8–40 mg/L to 4–20 mg/L was recommended by the AMS team based on the analysis. 

Corresponding to multiple other studies [12,36,37], the observed TA was found to be low. However, in our analysis the majority of samples (meropenem: 87.2%, piperacillin: 85.2%) were above the lower limit of the defined target range. This might indicate a focus of the prescribing intensive care physician on reaching effective drug exposures while at the same time accepting an increased risk of adverse events. Even if the upper limit of the target range and the toxicity thresholds used in this evaluation are based on very few clinical data and a confirmation via additional studies focusing on adverse drug reactions/toxicity is highly desired, no additional benefits of drug concentrations exceeding the PK/PD target of 100% *f*T_>4xMIC_ have been observed clinically [26,38,39,40,41]. Since higher antibiotic exposures seem not to increase efficacy but to increase the risk for adverse drug reactions/toxicity such as neurotoxicity (especially in renal failure), a reduction in doses should be considered. At the same time, the observed TA strongly varied between patients with different renal functions for both drugs; the highest proportion of TA was observed for patients with a normal renal function, while TA decreased both for worsening and elevated renal functions. This clearly indicated that the well-known impact of a patient’s individual renal function on the clearance of both antibiotics [12,42,43,44,45,46] had not been adequately taken into account for dosing decisions. Furthermore, the susceptibility of the causative pathogen or the empirical target (if the MIC was not determined) did not impact measured concentrations. Neither for meropenem nor for piperacillin did the observed drug concentrations differ significantly between patients with different targeted concentrations, indicating that the individual target range was not adequately considered during dosing decisions. In addition, the proportion of dosing adaptations after drug concentration measurement was found to be low (<10%) and the frequency of dosing adaptations after samples below the target range was not notably higher than after samples above the target range. For meropenem, the proportion of dose adjustments was found to be completely independent from TA, suggesting that the observed dose adaptations might not be due to the measured concentrations.

Overall, the real-word assessment revealed the need for actions on multiple levels to enable an optimal meropenem and piperacillin/tazobactam therapy: First, to ensure drug concentration targets sufficiently high enough to effectively treat local pathogens while at the same time avoiding unnecessary high drug burdens, the empirical targets should regularly be assessed based on the local susceptibility pattern [47]. Second, the renal function of the patient needs to be adequately taken into account [12,15,48]. Third, once additional information—i.e., MIC values or antibiotic concentration measurements—becomes available these need to be thoroughly analysed and, if required, lead to dosing adjustments [48]. As exemplified by our analysis at Charité-Universitaetsmedizin Berlin, current dosing is not adjusted based on the well-known link between the clearance of both drugs and the renal function of the individual patient. The observed data furthermore highlight the difficulty of integrating target information (e.g., infecting pathogen, MIC value) into individual initial dosing decisions. Adjusting dosing based on updated target information or individual drug measurements appeared to be an additional challenge. Evidently, the availability of information alone is not sufficient to encourage dose individualisation at the bedside. As seen in other antibiotics [49,50] and indications [51,52], meropenem and piperacillin dosing decisions need to be supported by clear and comprehensive dosing strategies developed and evaluated by the local AMS team. Dosing algorithms for initial antibiotic therapy integrating patient characteristics and target information will help to increase target attainment while standardising dosing. Clear guidelines on when and how to adjust dosing if new information becomes available, might increase the impact and benefit of a concentration monitoring program: De Waele et al. reported a substantial increase of target attainment when implementing a simple algorithm to adjust dosing after concentration measurements outside the target range [27]. Developed dosing algorithms [14,15,53,54] and interactive dosing tools (e.g., www.tdmx.eu [55] (accessed on 24 April 2022), www.dosing.de (accessed on 24 April 2022), www.thecaddy.de (accessed on 24 April 2022), www.clincalc.com (accessed on 24 April 2022)) could be employed to improve antibiotic dosing once the underlying PK models in those tools demonstrate appropriate predictive performance for the local patient population. Overall, a more structured and standardised approach to individualise antibiotic dosing at the bedside is needed to fully utilise all available information. 

The data presented and analysed in this manuscript were collected at two intensive care units at the Charité-Universitaetsmedizin Berlin and evaluated employing local targets selected by the AMS team. Specific *empirical* target should be identified individually in other institutions. Additionally, we recommend the continuous evaluation of the selected empirical targets based on local susceptibility data.

## 4. Materials and Methods

### 4.1. Study Design, Data Collection and Bioanalytical Method

This single-centre, prospective, observational study was carried out as part of a drug concentration monitoring program for meropenem and piperacillin at two intensive care units (Department of Infectious Diseases and Respiratory Medicine with 18 intensive care beds; Department of Surgery with 10 intensive care beds) at Charité-Universitaetsmedizin Berlin. The study protocol was approved by the local institutional review board (Charité Ethics Committee, application number: EA4/053/19). Patient’s data were included in the study if meropenem or piperacillin/tazobactam was administered as part of clinical practice to treat proven or suspected infections. Blood samples were taken based on the decision of the treating intensive care physician. Diagnosis, antibiotic dosing history prior to sampling, and clinical parameters including scores to assess the extent of organ function (e.g., SOFA score, creatinine clearance) were recorded. The available MIC values of relevant isolated pathogens were documented. 

Blood samples were collected at the end of the dosing interval (minimum drug concentration). Within one hour after sample collection, blood samples were sent to the laboratory, centrifuged, and stored at −20 °C until drug concentrations in plasma were determined by Labor Berlin (Labor Berlin-Charité Vivantes GmbH, Berlin, Germany) using high-performance liquid chromatography coupled with tandem mass spectrometry (Appendix B). The validation of the bioanalytical method revealed good analytical performance for both drugs (meropenem: calibration range 2–30 µg/mL, inaccuracy ≤ ± 5.9% relative error, imprecision ≤ 6.3% coefficient of variation; piperacillin: calibration range 20–120 µg/mL, inaccuracy < ± 5.3% relative error, imprecision ≤ 5.0% coefficient of variation). Concentrations outside the calibration range were diluted and re-evaluated. The results of the concentration measurement were available to the attending physician within 24 h after sampling. 

### 4.2. Pharmacokinetic/Pharmacodynamic Targets

Within the monitoring program, a single concentration measurement per patient and dosing interval was collected. To assess target attainment based on this single minimum drug concentration, a local target range for the minimum drug concentration was defined based on the available literature data [23,24,25,26,27,56] in consultation with the treating physicians and the AMS team at Charité-Universitätsmedizin Berlin: If the MIC value was available, the target range for unbound (=free) minimum meropenem and piperacillin concentrations was defined as 1–5 × MIC. For meropenem, MIC values below 1 mg/L were treated as 1 mg/L and for piperacillin MIC values below 4 mg/L were treated as 4 mg/L. If an MIC value could not be determined, the target range for unbound minimum concentrations was defined to be the highest MIC value still susceptible to either of the two antimicrobial drugs. Based on its anti-pseudomonal activity, the target range for meropenem was thus 8–40 mg/L (EUCAST breakpoint 8 mg/L) and 16–80 mg/L for piperacillin/tazobactam (EUCAST breakpoint 16 mg/L) [29]. This empirical target range for each of the two drugs was selected to account for the less susceptible pathogens expected at ICU wards [57,58]. Due to the low (~2%) protein binding of meropenem [59], the determined total concentrations were evaluated for meropenem while unbound piperacillin concentrations were calculated based on a literature reported fraction unbound (f_u_) of 91% in critically ill patients [60].

### 4.3. Antimicrobial Treatment 

Antibiotic dosing prior to and dose adjustments after concentration measurements were determined by the attending physician based on the patient’s clinical condition. No structured procedure for dose adjustments following concentration measurements outside the target range was pre-specified. For all prolonged or continuous infusions, a loading dose (0.5 h infusion) of 1 g meropenem or 4 g piperacillin/0.5 g tazobactam was recommended by the AMS team. Pathogens with MIC values above the EUCAST breakpoint for anti-pseudomonal activity of the investigated drugs (meropenem: 8 mg/L, piperacillin/tazobactam: 16 mg/L) were defined as resistant and a change of antibiotic was recommended to the attending physician. 

### 4.4. Data Analysis

Statistical measures used to characterise the central tendency and dispersion of distributions (median, percentiles, and range) were calculated for continuous data. All statistical analyses were performed in R/RStudio (v.3.6.3/1.3.959) using the packages (ggplot2 (v.3.3.1), dplyr (v.1.0.0), stats (v.3.6.3)).

### 4.5. Target Attainment Assessment

The proportion of measured concentrations in the target range was determined for both drugs depending on the availability of microbiological data (i.e., the target range either 1–5 × MIC or in empirical therapy if no MIC is available 8–40 mg/L for meropenem and 16–80 mg/L for piperacillin/tazobactam). Furthermore, the proportion of drug concentrations below drug concentrations linked to a reported increase in neurotoxicity corresponding to 64 mg/L for meropenem [38] and 157 mg/L for piperacillin in combination with tazobactam [39], respectively, was calculated.

### 4.6. Target Attainment Assessment in Different Renal Function Groups

To assess if TA was independent of patients’ renal function, target attainment was stratified by renal function (assessed by creatinine clearance estimated based on the Cockcroft–Gault formula [33]: severe renal impairment (RI) 0– 30 mL/min, moderate RI >30–60 mL/min, mild RI > 60–90 mL/min, normal renal function (RF) > 90–130 mL/min, augmented RF > 130 mL/min) [61] and subjected to a chi-squared test of independence. A significant difference in target attainment between renal function groups (*p*-value < 0.01) would indicate lack of evidence for adequately adjusted dosing based on renal function. 

### 4.7. Drug Concentration Assessment between Different Target Range Groups

If higher individual MIC values and therefore higher antibiotic concentrations to be targeted were adequately incorporated into dosing decisions, samples with higher targets should display higher drug concentrations. The Kruskal–Wallis test was used to assess if measured antibiotic drug concentrations were significantly higher for patients with higher targets. A non-significant difference between different targeted concentrations (*p*-value > 0.01) would indicate lack of evidence for adequately adjusted dosing based on the targeted concentration range of the individual patient.

### 4.8. Dosing Adaptations

The frequency and nature of dosing adaptations after measured concentrations below, in, and above the target range was assessed. To investigate if dosing adaptations after measured concentrations outside the target range were more frequent than dose adaptations after measured concentrations inside the target range, the Kruskal–Wallis test was used.

## 5. Conclusions

The high empirical targets in comparison to the observed local susceptibility data presented in this evaluation clearly illustrate the need for a regular assessment of empirical targets. Furthermore, the suboptimal target attainment and the low number of dosing adaptations after concentration measurements outside the target range, calls for the translation of scientific knowledge into easy-to-use dosing strategies guiding antibiotic dosing.

## Figures and Tables

**Table 1:** Patient and sampling characteristics.

Characteristic	Meropenem	Piperacillin/Tazobactam
**Patient level**		
**Categorical**	***n*** **(%)**	***n*** **(%)**
Patients	108	96.0
Male	70.0 (64.8)	61.0 (63.5)
**Continuous [unit]**	**Median (5th–95th percentile)**	**Median (5th–95th percentile)**
Age (years)	62.0 (36.0–80.0)	65.0 (36.081.0)
Weight (kg)	76.0 (49.0–126.0)	76.0 (49.6–128.3)
**Sample level**		
**Categorical**	***n*** **(%)**	***n*** **(%)**
Samples	375	230
Samples during CRRT	143 (38.1)	53 (23.0)
Samples during ECMO	33 (8.80)	12 (5.22)
Location of infection:		
Intraabdominal	166 (44.6)	101 (43.9)
Pneumonia	131 (35.2)	102 (44.3)
Blood stream	60 (16.1)	18 (7.83)
Skin-/soft tissue	15 (4.00)	4 (1.74)
Other	0 (0)	5 (2.17)
Unknown	3 (0.80)	0 (0)
**Continuous [unit]**	**Median (5th–95th percentile)**	**Median (5th–95th percentile)**
Samples per patient	2.00 (1.00–9.60)	2.00 (1.00–5.20)
Creatinine clearance ^#^ (mL/min)	76.6 (24.8–241)	71.1 (17.8–171)
Serum albumin conc. (g/dL)	2.68 (1.99–3.58)	2.70 (2.00–3.50)
SOFA score	8.00 (1.90–17.0)	6.00 (1.00–14.0)
APACHE score	23.0 (12.0–37.0)	20.0 (11.0–34.0)

^#^ estimated according to Cockcroft–Gault formula [33]. Abbreviations: *n*: number, conc.: concentration, CRRT: continuous renal replacement therapy, ECMO: extracorporeal membrane oxygenation, SOFA: Sepsis-related organ failure assessment score, APACHE: Acute physiology and chronic health evaluation score.

**Table 2:** Observed minimum inhibitory concentration (MIC) values.

	Meropenem *n* (%)	Piperacillin/Tazobactam *n* (%)
Patients with determined MIC	53 (49.1)	33 (34.4)
Unique MIC determinations	60	33
MIC values (mg/L):		
≤0.25	45 (75.0 *)	2 (6.06 *)
0.5	2 (3.33 *)	-
1	2 (3.33 *)	-
2	2 (3.33 *)	-
4	3 (5.00 *)	24 (72.7 *)
8	2 (3.33 *)	5 (15.2 *)
16	4 (6.67 *)	1 (3.03 *)
32	-	1 (3.03 *)

Abbreviations: MIC: minimum inhibitory concentration, *n*: number. * in relation to the number of unique MIC determinations (*n* = 60 for meropenem, *n* = 33 for piperacillin; if a second MIC determination in the same patient was equal to the first determination it was not considered in this table).

**Table 3:** Overview of observed adaptations in the dosing regimen for meropenem and piperacillin.

Samples	Dosing Adaptations, *n* (%)			
	All	Below Target Range	In Target Range	Above Target Range
**Meropenem**				
Total	22 (6.18 *)	3 (6.12 *)	7 (5.51 *)	12 (6.67 *)
Dose reduction	7 (31.8 ^#^)	0 (0 ^#^)	0 (0 ^#^)	7 (58.0 ^#^)
Dose increase	8 (36.4 ^#^)	3 (100 ^#^)	3 (43.0 ^#^)	2 (17.0 ^#^)
Dosing interval reduction	2 (9.09 ^#^)	0 (0 ^#^)	1 (14.0 ^#^)	1 (8.3 ^#^)
Dosing interval increase	2 (9.09 ^#^)	0 (0 ^#^)	1 (14.0 ^#^)	1 (8.3 ^#^)
Infusion duration reduction	2 (9.09 ^#^)	0 (0 ^#^)	1 (14.0 ^#^)	1 (8.3 ^#^)
Infusion duration increase	1 (4.54 ^#^)	0 (0 ^#^)	1 (14.0 ^#^)	0 (0 ^#^)
**Piperacillin**				
Total	10 (4.63 *)	4 (12.5 *)	0 (0 *)	6 (8.00 *)
Dose reduction	0 (0 ^#^)	0 (0 ^#^)	0 (0 ^#^)	0 (0 ^#^)
Dose increase	0 (0 ^#^)	0 (0 ^#^)	0 (0 ^#^)	0 (0 ^#^)
Interval reduction	4 (40.0 ^#^)	3 (75.0 ^#^)	0 (0 ^#^)	1 (17.0 ^#^)
Interval increase	5 (50.0 ^#^)	0 (0 ^#^)	0 (0 ^#^)	5 (83.0 ^#^)
Infusion duration reduction	1 (10.0 ^#^)	1 (25.0 ^#^)	0 (0 ^#^)	0 (0 ^#^)
Infusion duration increase	0 (0 ^#^)	0 (0 ^#^)	0 (0 ^#^)	0 (0 ^#^)

* in relation to the number of determined drug concentrations per column and antibiotic, ^#^ in relation to the total number of dose adaptations. Abbreviations: *n*: number of samples.

## Data Availability

The data presented in the study are available at reasonable request from the corresponding author.

## Appendix B

### Instrumentation and LC-MS/MS Conditions

The LC-MS/MS system (HPLC: Shimadzu Deutschland GmbH, Duisburg, Germany, MS: Sciex LLC, Framingham, MA 01701, USA) was used. The Prominence 20 LC system consisted of a multisampler, set at 4 °C, injection volume 10 µL, a high-speed pump and a multicolumn thermostat (set at 60 °C). A Phenomenex Kinetex C 8; 2.6 μm; 100 A; 75 mm × 3 mm with a suitable precolumn (Phenomenex C 8; 5 µm; 4.0 mm × 3.0 mm) was used for chromatographic separation. For elution, a gradient of ultra-pure water + 0.2% formic acid [*v*/*v*] (Eluent A) and methanol (Eluent B) was used with a flow rate of 0.65 mL/min. The final gradient is shown in Table A1. The total run time was 3.7 min. The LC system was coupled to a triple quadrupole MS/MS system (Sciex QTRAP 5500) with electrospray ionisation (ESI) source and operated in positive ionisation mode. Ion acquisition was performed using multiple reaction monitoring (MRM) including the transitions for meropenem and piperacillin shown in Table A2. The optimised ion source parameters are shown in Table A3. Data acquisition and processing was carried out using Sciex LLC, Framingham, MA, USA.

**Table A1:** Gradient elution for quantification of meropenem and piperacillin.

Time [min]	Eluent A [%]	Eluent B [%]
0.01	99	1
0.35	45	55
1.30	45	55
1.65	1	99
2.10	1	99
2.70	99	1
3.70	99	1

Mobile phase A: ultra-pure water + 0.2% formic acid [*v*/*v*], Mobile phase B: methanol.

**Table A2:** Precursor ions *m*/*z* and product ions *m*/*z* of meropenem and piperacillin with corresponding applied mass spectrometric parameters of the developed LC-MS/MS method.

Compound	Precursor Ion *m*/*z*	Product Ion *m*/*z*	Dwell Time [ms]	Collision Energy [V]	Declustering Potential [V]	Entrance Potential [V]	Collision Cell Exit Potential [V]
Meropenem 1	384.1	**340.2**	30	11	231	10	24
Meropenem 2	384.1	298.3	20	21	1	10	24
Piperacillin 1	518.2	**160.2**	30	9	280	10	22
Piperacillin 2	518.2	143.3	30	51	191	10	16

Transition used for quantification marked in bold.

**Table A3:** Ion source parameters of the mass spectrometer.

Curtain Gas [psi]	Collision Gas [psi]	Ionspray Voltage [V]	Temperature of Ion Source [°C]	Nebulizing Gas [psi]	Drying Gas [psi]
40	9	4000	500	45	70
